# Microbial biotechnology for the synthesis of (pro)vitamins, biopigments and antioxidants: challenges and opportunities

**DOI:** 10.1111/1751-7915.12379

**Published:** 2016-07-04

**Authors:** Jose L. Revuelta, Ruben M. Buey, Rodrigo Ledesma‐Amaro, Erick J. Vandamme

**Affiliations:** ^1^ Department of Microbiology and GeneticsFaculty of BiologyUniversidad de SalamancaSalamancaSpain; ^2^ Department of Biochemical and Microbial TechnologyFaculty Bioscience EngineeringGhent UniversityGhentBelgium

## Abstract

Vitamins and related compounds, such as provitamins, biopigments and antioxidants, belong to those few chemicals that appeal in a positive way to most people. These terms sound synonymous to vitality, good health and mental strenght, even to the layman. Everyone of us needs his/her daily intake of (pro)vitamins and antioxidants, normally provided by a balanced and varied diet. However, current food habits or preferences, food availabilities, as well as food processing, preservation or cooking methodologies and technologies, do not always assure a sufficient balanced natural daily (pro)vitamin supply to a healthy individual, and even more so for a stressed or sick human being. Today, modern society is seldom confronted with the notorious avitaminoses of the past, well known to the Western World, but they do still occur frequently in overpopulated, war‐ridden, poverty‐ or famine‐struck regions on our globe, as well as for surprisingly large population groups in developed countries.

## Need for efficient bio‐production systems for vitamin, biopigments, antioxidants and related health factors

Vitamins and related compounds, such as provitamins, biopigments and antioxidants, belong to those few chemicals that appeal in a positive way to most people. These terms sound synonymous to vitality, good health and mental strenght, even to the layman. Everyone of us needs his/her daily intake of (pro)vitamins and antioxidants, normally provided by a balanced and varied diet. However, current food habits or preferences, food availabilities, as well as food processing, preservation or cooking methodologies and technologies, do not always assure a sufficient balanced natural daily (pro)vitamin supply to a healthy individual, and even more so for a stressed or sick human being. Today, modern society is seldom confronted with the notorious avitaminoses of the past, well known to the Western World, but they do still occur frequently in overpopulated, war‐ridden, poverty‐ or famine‐struck regions on our globe, as well as for surprisingly large population groups in developed countries. Apart from their in vivo nutritional‐physiological roles as essential growth factors and coenzymes for man, animals, plants and microorganisms, vitamins and related compounds are increasingly being introduced as food and as feed additives, as medical‐therapeutical agents, as health promoting aids. Nowadays an impressive number of processed foods, feeds, cosmetics, pharmaceutical and chemical formulations contain extra‐added (pro)vitamins or vitamin‐related compounds, and single and multivitamin preparations are commonly taken or prescribed.

In addition to their well known nutritional, physiological and medical importance, (pro)vitamins and related health compounds have also found large‐scale technical applications, for example, as antioxidants (D‐isoascorbic acid as the C5‐epimer of vitamin C, glutathione or GSH, tocoferol or vitamin E, carotenoids, wine and tea polyphenols), as acidulants (ascorbic acid or vitamin C) and as bio‐pigments (yellow‐orange‐red carotenoids, yellow riboflavin or vitamin B_2_, red *Monascus*‐pigments) in the food, feed, cosmetic, chemical, nutraceutical and pharmaceutical sectors. There is especially a need for natural pigments of (micro)biological origin to replace synthetic pigments and colourants. Certain fungal carotenoids (*Blakeslea trispora* – beta‐carotene, *Xanthophyllomyces dendrorhous* – asthaxanthin) and algal carotenoids (*Dunaliella salina –* xanthophylls, lycopene), cochineal‐carminic acid from scale insects, blue‐purple phycocyanin from *Arthrospira*‐cyanobacteria, and fungal dark reddish monascin‐pigments are already used in this respect, but these bioprocesses need to be further improved as to yield and biotechnology tools involved (Vandamme, 2002, 2011; Babitha, 2009; Yoshida *et al*., 2009; Patakova, 2013; Vandamme and Revuelta, 2016). The above considerations point towards an extra need for (bio)synthesis and supply of (pro)vitamin, biopigment, antioxidant and related health molecules, above the level provided naturally from microbial, plant and animal food sources.

## Switch from extraction technology over chemical synthesis towards industrial biotechnology‐based processes

Till a few decades ago most added vitamins and related health compounds were indeed industrially prepared via extraction technologies. Concentrates or extracts derived from vitamin‐rich or coloured natural staple food products (of plant, animal or microbial origin), however, find now relatively little use in the food, feed, pharmaceutical or cosmetic sector. Apart from their high price, some of the reasons are:

- the level of vitamins and related health compounds in the natural plant/animal source is usually relatively low and fluctuates drastically (i.e. exceptions are essential fatty acids or EFA's (also known as PUFA's) in plant oils and fish oils, vitamin D in fish oils).
- their organoleptic presentation and shelf ‐life is often not optimal.
- water‐soluble vitamins are easily lost by aqueous extraction or other manipulations of these natural food vitamin sources
- (pro)vitamins and related health compounds are labile molecules during the process of harvest, preservation, storage (or during preparation of foodstuffs) and are generally sensitive to pH, heat (riboflavin or B_2_, D‐pantothenic acid or B_5_, pyridoxine or B_6_, folic acid or B_9_, vitamin C, vitamin E), light (B_2_, B_6_, B_9_, vitamin B_12_, C, vitamin D), oxygen (B_9_, C, D, essential fatty acids or EFA's).

These drawbacks have led to the industrial manufacturing of most vitamins and related factors by chemical or microbial synthesis routes. Currently, several vitamins are made chemically (pro‐vitamin A, cholecalciferol or D_3_, E, vitamin K_1_ or phylloquinone and thiamine or B_1_, B_5_, B_6_, D‐biotin or B_7_, B_9_), although enzymatic, microbiological and/or biotechnological methods emerge or exist, although not economically profitable as yet (Demain, 2000, 2007; Laudert and Hohmann, 2011). For some of these molecules or their precursors, biotechnological processes are being developed, although indeed not competitive as yet with chemical synthesis.

Two biotechnological routes, directed fermentation processes and biocatalysis, take gradually over from chemical synthesis for most of these chemically complex molecules. Both technologies were initially often rescued only when chemical processes failed to be successful or were uneconomical. Nowadays they become often first choice technologies for several reasons: they are based on renewable resources, deliver simple as well as very complex molecules directly in a desirable chiral form and in an economically favourable way and they are considered in society as clean, sustainable and reuse technologies.

Vitamin and vitamin‐like compounds that are produced (exclusively) by microbial fermentation with bacteria, yeasts or fungi include vitamin C, B_2_, B_12_, and ergocalciferol or D_2,_ EFA's, menaquinone or K_2_, coenzyme Q_10_ or ubiquinone, pyrrolquinoline quinine or PQQ. The antioxidant glutathione (GSH) is currently produced with the yeasts *Saccharomyces cerevisiae* or *Candida utilis* (Li *et al*., 2004; Wang *et al*., 2016), while the health supplement gamma‐aminobutyric acid (GABA) is produced based on a two‐step bioprocess: glutamate fermentation with *Corynebacterium*, and subsequent conversion into GABA with lactic acid bacteria‐derived overexpressed glutamate decarboxylase (Shi and Li, 2011; Pham *et al*., 2016). Some molecules can be produced by a combination of chemical steps and microbial/enzymatic steps (niacin or B_3_, B_5_, C, L‐carnitine) (Vandamme, 1989, 1992; De Baets *et al*., 2000; Shimizu, 2008; Laudert and Hohmann, 2011; Eggersdorfer *et al*., 2012; Vandamme and Revuelta, 2016). Some are produced via microalgal culture in ponds or fermentor vessels (beta‐carotene, EFA's) (Cadoret *et al*., 2012; Borowitzka, 2013).

## Challenges and future developments

The detailed biosynthesis pathways (and their metabolic regulation and controls) used by those microorganisms have been elucidated for several (pro)vitamins, biopigments, antioxidants and similar health compounds, but this was only realized gradually over the last decades, mainly by studying model microbial strains and/or producer microorganisms, such as bacteria (*Escherichia coli*,* Serratia*,* Bacillus*,* Lactobacillus*,* Pseudomonas*,* Gluconobacter*,* Sinorhizobium*,* Agrobacterium*,* Hyphomicrobium*,* Propionibacterium*,* Rhodobacter*,* Rhodococcus*,* Arthrospira‐*cyanobacteria*)*, yeasts (*Saccharomyces*,* Candida*,* Xanthophyllomyces*,* Yarrowia*), fungi (*Blakeslea*,* Ashbya*,* Mortierella*,* Mucor*,* Monascus*), as well as green microalgae (*Dunaliella*,* Euglena*,* Haematococcus*), marine non‐photosynthetic dinoflagellates (*Crypthecodinium)* and marine non‐photosynthetic Thraustochytrid‐microalgae (*Schizochytrium*) (Laudert and Hohmann, 2011; Borowitzka, 2013; Ledesma‐Amaro *et al*., 2013; Bellou *et al*., 2014; Vandamme and Revuelta, 2016). It turned out that the pathways involved and their metabolic regulations are very complex and often very difficult to deregulate to arrive at overproduction levels of the desired compounds.

For some of the vitamins, biopigments and antioxidants and other health factors, microbial overproduction to reach industrially relevant levels still remains a challenge. For GABA production, a high‐yielding one‐step fermentation process directly from glucose should replace the currently two‐stage process now in use. In the case of biotin or B_7_, efficient bioconversion of dethiobiotin to biotin (the last biochemical step in the pathway) remains a real scientific bottleneck. As to vitamin B_1_ and B_9,_ highly engineered *Bacillus subtilis* strains converted expensive precursors only into quite low B_1_ or B_9_ levels, respectively, preventing industrial application so far. For vitamin C, a defined mixed co‐culture process is recently established on a large scale, and even a direct fermentation route is about to bring a breakthrough. Furthermore, it is well known that gut microbiota, mainly anaerobic eubacteria and archaea, act as vitamin suppliers to their hosts (Le Blanc *et al*., 2013); however, few of them have been studied sufficiently in this context. They could be a source of novel genes and for strain development for industrial vitamin and other growth and health factor production.

Apart from obtaining these vitamins, biopigments, antioxidants and related compounds via a microbial process – what microbial fermentation, biocatalysis and algal culture is all about –, fermentation‐based or enzymatic biocatalytic processes furthermore yield the desired enantiomeric compound, and they can be redirected via genetic and biotechnological modification of the involved bacteria, yeast and fungi or microalgae into high‐yielding production systems.

**Figure 1:**
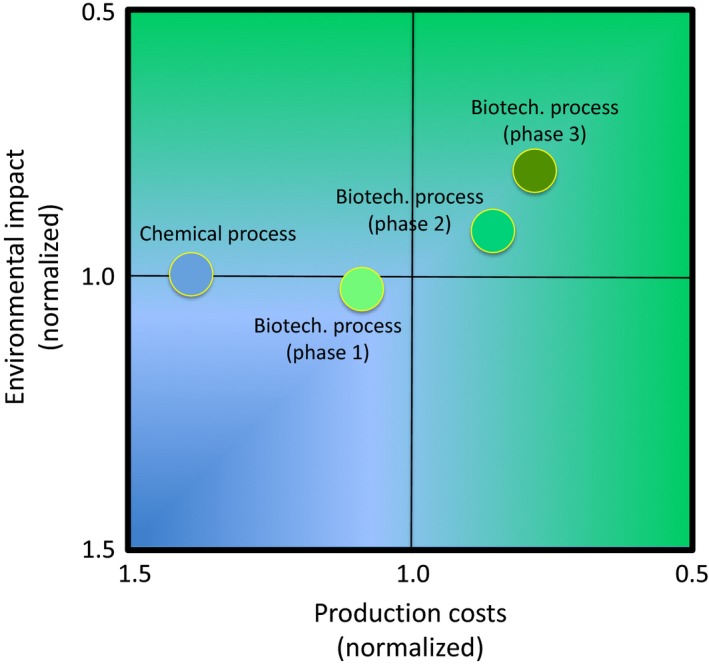
Environmental and economic impact analysis of riboflavin (vitamin B_2_) chemical and biotechnological processes in different phases of development (phase 1, 2 and 3).

Scientific breakthroughs in high‐throughput screening methodologies, in molecular genetics of industrial microbial strains, in systems (micro)biology, in directed evolution, metabolic engineering and modelling, but equally in enzyme and cell engineering has allowed to make progress towards industrial realizations of vitamin and vitamin‐like compounds production. A striking example is the production by fermentation of vitamin B_2_ using *Ashbya gossypii*. The productivity of the initial industrial strains developed by classical mutagenesis techniques has been greatly improved by a combination of metabolic engineering, comparative genome and transcriptome analysis and genome metabolic modelling approaches (Karos *et al*., 2004; Ledesma‐Amaro *et al*., 2014, 2015). The success of this strain improvement programme allowed the replacement at industrial level of the vitamin B_2_ chemical process by the currently used based on microbial fermentation. The implementation of the biotechnological process reduced the production and environmental protection costs by 43% in relation to the chemical manufacturing process (Fig. 1). Furthermore, CO_2_ emission was reduced by around 30%. As result of its single main step, fermentation has substantial cost savings compared with the multi‐stage chemical process (Wenda *et al*., 2011).

Also, combination of directed evolution and rational protein design using computational tools has become significant to create even novel enzymes, expanding their application potential in the field of (pro)vitamin and related fine chemical biosynthesis. Asymmetric biocatalysis with microbial enzymes and cells has now achieved high efficiency, enantioselectivity and yield, such that – for a wide variety of chiral products, including (pro)vitamins, biopigments, antioxidants and related compounds – biocatalysis has become a preferred production alternative in organic synthesis and in the (bio)chemical industry for fine as well as for bulk chemicals.

Novel culture techniques, rapid sampling and sensor methodologies, improved bioreactor design and downstream processing, all will contribute to the growing interest to use industrial microbiology‐ and biotechnology‐based processes in industry. The design‐based engineering of industrial microbial strains is still hampered by incomplete knowledge of cell biochemistry, metabolic regulation and cell biology. Advances in systems biology technologies and in synthetic (micro)biology can now also contribute to fill this gap. Especially a mix of all these advancements will allow for high‐yielding microbial strains to be constructed that are suitable for industrial microbial‐based production of (pro)vitamins, biopigments, antioxidants and related health compounds. In the future, the advent of synthetic biology will further lead to the tailor made construction of high‐yielding microbial (pro)vitamin, biopigment, antioxidant and other health factor producer strains.

## Conflict of interest

The authors declare that they have no competing interests.
